# Melatonin improves age-induced fertility decline and attenuates ovarian mitochondrial oxidative stress in mice

**DOI:** 10.1038/srep35165

**Published:** 2016-10-12

**Authors:** Chao Song, Wei Peng, Songna Yin, Jiamin Zhao, Beibei Fu, Jingcheng Zhang, Tingchao Mao, Haibo Wu, Yong Zhang

**Affiliations:** 1 College of Veterinary Medicine, Northwest A&F University, Yangling 712100, Shaanxi, China; 2 Key Laboratory of Animal Biotechnology, Ministry of Agriculture, Northwest A&F University, Yangling 712100, Shaanxi, China

## Abstract

Increasing evidence shows that melatonin protected against age-related mitochondrial oxidative damage. However, the protective effects of melatonin against ovarian aging has not been explored. Young Kunming females (aged 2–3 months) were fed with melatonin added to drinking water for 6 or 12 months (mo). We found that long-term (12 mo) melatonin treatment significantly reduced ovarian aging, as indicated by substantial increases in litter size, pool of follicles, and telomere length as well as oocyte quantity and quality. Melatonin treatment suppressed ovarian mitochondrial oxidative damage by decreasing mitochondrial reactive oxygen species (mROS) generation, inhibiting apoptosis, repressing collapse of mitochondrial membrane potential and preserving respiratory chain complex activities. Female mice fed with melatonin had enhanced mitochondrial antioxidant activities, thus reducing the risk of mitochondrial oxidative damage cause by free radicals. Notably, melatonin treatment enhanced SIRT3 activity but not the protein expression level, and increased the binding affinity of FoxO3a to the promoters of both superoxide dismutase 2 (SOD2) and catalase (CAT). In conclusion, melatonin exerted protection against aging-induced fertility decline and maintenance of mitochondrial redox balance.

Female fertility peaks at approximately age 25 years and declines rapidly after age 35 years^1^. However, with current cultural and social trends, many women around the world delay childbearing and many face infertility when they attempt to conceive because of the ovarian aging, rather than changes in endometrial receptivity^2^. Ovarian aging is accompanied by a significant decline in telomere length^3^, ovarian follicle pool and oocyte reserve^4^, as well as an increased number of low-quality oocytes not competent for further development^5^,^6^. Thus, reproductive senescence is a growing public health problem.

Mitochondria are the primary energy generators in the body and are also the major source and target of free radicals^7^. In particular, mitochondrial oxidative stress is considered a major factor contributing to the aging process and leads to aberrant signaling pathways^8^,^9^. Aging increased mitochondrial reactive oxygen (mROS) and weakened antioxidative defense systems^10^. Therefore, it is necessary to attenuate oxidative stress and strengthen the antioxidant system to modulate altered signaling pathway molecules to constrain the aging process.

Melatonin (N-acetyl-5-methoxytryptamine) is an endogenously produced indoleamine that can modulate many important physiological reactions in the body^11^. Long-term administration of melatonin, whose pineal secretion decreases with age, was highly effectively at preventing age-dependent impairment of redox status in various tissues and cells^12^. Melatonin modulates mitochondria-related functions and strengthens its antioxidant defense systems, effects facilitated by its amphiphilic nature, enabling it to penetrate all morphophysiological barriers and enter all subcellular compartments. Furthermore, melatonin and its metabolites were powerful antioxidants and free radical scavengers^13^,^14^. This property enabled them to protect cellular membranes, the electron transport chain and mitochondria from oxidative injury. Melatonin would be readily bioavailable to the ovary and could directly affect ovarian function after oral ingestion^15^. Thus, the use of melatonin to improve age-related mitochondrial oxidative stress in the ovaries seems feasible.

The aims of this study were to determine whether mice receiving long-term treatment with melatonin could counteract age-associated infertility and ovarian mitochondrial oxidative stress in a mouse model of ovarian aging *in vivo* and *in vitro*.

## Materials and Methods

### Ethics Statement

This study was carried out in strict accordance with the guidelines for the care and use of animals of Northwest A&F University. All animal experimental procedures were approved by the Animal Care Commission of the College of Veterinary Medicine, Northwest A&F University. Every effort was made to minimize animal pain, suffering, and distress and to reduce the number of animals used.

### Reagents

Unless otherwise stated, all chemicals and reagents used in this study were from Sigma-Aldrich Chemical Co. (St. Louis, MO, USA).

### Animals and treatments

Female Kunming strain mice at 2–3 mo old (outbreed albino mice originated from the ICR strain) were obtained from the Experimental Animal Center of the Fourth Military Medical University (Xi’an, China) and housed in a temperature-controlled animal facility, kept on a cycle of 12 h light and 12 h dark with free access to food and water. Mice were randomly divided into two groups: half received vehicle as a control and the other half received melatonin at 10 mg/kg of body weight during the hours of darkness (18:00–06:00). The dose of melatonin was selected based on that used in previous aging studies in mice^12^,^16^. Melatonin was dissolved in a minimum volume of absolute ethanol plus tap water to yield a dose of 10 mg/kg of body weight. The concentration of ethanol in the final solution was 0.066%. A fresh vehicle and melatonin solutions were prepared twice per week. Water bottles were covered with aluminum foil to protect solutions from light. Following treatment with melatonin or vehicle for 6–12 months. Female mice of different age group were paired with young adult Kunming males of proven fertility at 22:00 h. Successfully mated were confirmed by the presence of a vaginal plug before 10:00 h the following morning (day 1 of pregnancy) and were bred individually. Pregnant time of each mouse, number of pups in one litter and development of pups were recorded.

### Determination of melatonin levels

Melatonin levels were determined from plasma samples that were collected from each groups. The mice were decapitated after 20% urethane solution. Blood was collected by cardiac puncture, five hundred microliters of were collected in a small-heparinized and kept on ice. The blood was centrifuged at 1,500 rpm for 20 min at 4 °C. Plasma was kept at −80 °C until analysis. Melatonin levels were determined taking all the necessary care to prevent light degradation by using the melatonin enzyme immunoassay kit (IBL Hamburg, Germany).

### Ovary serial sectioning and follicle counting

Ovaries were collected and fixed in 4% paraformaldehyde for 24 h, then paraffin embedded. Serial sections (5-μm thickness) were cut and placed onto glass microscope slides. After staining with hematoxylin and eosin Y, the stained sections were analyzed for the number of follicles at four different developmental stages under an optical microscope (Nikon U-III Multipoint Sensor System, Japan). The follicles were classified as previously described^17^.

### Estimation of mitochondrial DNA (mtDNA) copy numbers

Oocyte manipulation techniques are described in more detail in Supplemental materials. A single GV or MII oocyte was added to a PCR tube with 20 μL of lysis buffer (50 mM Tris, 0.1 mM EDTA, 100 μg/mL Proteinase K and 0.5% Tween-20) and incubated at 55 °C for 30 min, then 95 °C for 10 min. To obtain purified plasmid standard DNA, PCR products were amplified with a mouse mtDNA-specific primer as previously described^18^, and were ligated into PMD-19T vector (Takara).

### Immunofluorescence

For chromosome and spindle analysis, oocytes from each group were washed twice in PBS containing 0.1% polyvinylpyrrolidone (PBS-PVP) and then fixed with freshly prepared 4% paraformaldehyde on ice for 30 min, permeabilized with 0.1% Triton X-100 for 30 min and washed three times. After blocking in 1% Bull Serum Albumin (BSA)-supplemented PBS for 1 h, oocytes were incubated overnight at 4 °C with fluorescein isothiocyanate-conjugated α-tubulin antibody to visualize microtubules in the spindle. Chromosomes were stained with DAPI for 5 min. Embryos were harvested for the detection of Oct4-positive cells with an anti-Oct4 mouse monoclonal antibody (Santa Cruz, CA, USA). DNA fragmentation was assessed in blastocysts using a DeadEnd Fluorometric TUNEL System (Promega, Madison, WI), according to the manuscript’s instructions.

### Telomerase activity assay

The telomerase activity in ovaries of was measured using the telomerase (TE) ELISA kit (CUSABIO, Wuhan, China).

### Measurement of average telomere length

DNA was extracted from ovaries using TIAN amp Genomic DNA Kit (Tian Gen Biotech CO., Ltd., Beijing, China) as described by the manufacturer and absorbance at 260 nm measured with a spectrophotometric plate reader (BioTek, Winooski, Vermont, USA). Average telomere length was measured from genomic DNA using a quantitative real-time PCR (qRT-PCR) assay. The telomere signal was normalized to that from the reference control gene to generate a T/S ratio indicative of relative telomere length.

### Mitochondrial ROS production assay

Mitochondrial ROS production was determined using the CM-H_2_DCFDA indicator dye (Molecular Probes; Invitrogen, Eugene, OR, USA)^19^.

### Mitochondrial DNA damage

Samples of mitochondrial DNA (1 mg/mL) were isolated using Mitochondrial DNA Isolation Kit (BioVision, Milpitas, CA, USA) and incubated for 5 min at 95 °C, then rapidly chilled on ice. DNA sample were digested to nucleosides by incubating the denatured DNA with 3 μL sodium acetate (20 mM, pH 5.2) and 2 μL of nuclease P1 (6.25 U/μL) for 2 h at 37 °C. Each sample was then incubated with 2 μL alkaline phosphatase (0.31 U/μL) and 6 μL Tris (100 mM, pH 7.5) for 1 h at 37 °C. The reaction mixtures were then centrifuged for 5 min at 6000 × g and the supernatants assayed for DNA oxidation with an 8-hydroxy-2′-deoxyguanosine (8-OHdG) ELISA assay (Cell Biolabs, CA, USA).

### Measurement of mitochondrial antioxidant function

The mitochondrial fractions were assayed for the superoxide dismutase 2 (SOD2), catalase (CAT) and glutathione (GSH/GSSG) activities. All activities were measured using the kit, following the manufacturer’s instructions (Beyotime Institute of Biotechnology, Jiangsu, China).

### Isolation of cytosolic, mitochondria and nuclear fractions

Mitochondria or nuclear fractions were immediately extracted using the Mitochondrial or Nuclear Isolation Kit (Beyotime), respectively. All manipulations were performed on ice. Protein concentrations were determined with the BCA Protein Assay Kit (Pierce Biotech, Rockford, IL, USA).

### Western blotting

Protein samples were separated by gradient electrophoresis on 8–15% SDS/PAGE gels. The following antibodies were used to detect the proteins of interest: Glyceraldehyde-3-phosphate dehydrogenase (GAPDH), LAMIN A, β-TUBULIN, SIRT3, Cytochrome c, Bax, Bcl-2, and Cytochrome c oxidase (COX) IV from Cell Signaling Technology (Danvers, MA, USA) and FoxO3a, SOD2, CAT from Abcam (Cambridge, UK). Western blot analysis was performed as described previously^20^ using the protocols provided by the primary antibody suppliers.

### Determination of adenosine triphosphate (ATP) content

The levels of ATP were measured with a luminometer (Bioluminat Junior, Berthold, Germany) with an ATP Bioluminescence Assay Kit (Beyotime).

### Ovarian mitochondrial membrane potential (MMP) and respiratory chain complex activity (I-IV) measurements

Before analysis, mitochondria samples were subjected to three freeze-thaw cycles to disrupt membranes and expose the enzymes. All enzymatic activities were measured at 37 °C. MMP was determined using JC-1 as previously described^21^. Complex I (NADH-ubiquinone oxidoreductase) activity was assayed by measuring decreased absorbance at 340 nm, indicating reduction of NADH oxidation. Complex II (succinate-ubiquinone oxidoreductase) activity was assayed by monitoring reduction of 2,6-dichlorophenolindophenol (DCPIP) at 600 nm. Complex III (CoQ-cytochrome c oxidoreductase) was assayed by monitoring reduction of cytochrome c at 550 nm. Complex IV (cytochrome c oxidase) was assayed by monitoring the oxidation of reduced cytochrome at 550 nm^16^.

### Mouse primary granulosa cell collection and culture

Mouse granulosa cells were collected from the ovaries of immature (3 weeks) Kunming mice using the follicle puncture method, as described previously^22^. The granulosa cells were cultured in DMEM/F12 supplemented with 10% FBS, 100 IU/mL penicillin, and 100 μg/mL streptomycin at 37 °C and 5% CO_2_. Cells were incubated with 2 mM H_2_O_2_ in the presence or absence of melatonin (100 μg/mL) for 2 h. After incubation, cells were used for further analysis.

### Cell viability assay

Cell viability was evaluated by the Cell Counting Kit-8 (CCK-8, Beyotime). After treatment, 10 μL CCK-8 solution was added to each well and incubated at 37 °C for 4 h.

### Apoptosis analysis by flow cytometry

Cell suspension was treated with 5 μL of Annexin V and 2.5 μL of propidium iodide (Beyotime) in the dark for 15 min, and analyzed by flow cytometry using a FACSCalibur flow cytometer (Becton-Dickinson, San Jose, CA, USA).

### SIRT3 activity

Protein was extracted from cells and tissue using a mild lysis buffer (50 mM Tris-HCl pH 8, 125 mM NaCl, 1 mM DTT, 5 mM MgCl_2_, 1 mM EDTA, 10% glycerol, and 0.1% NP-40). SIRT3 activity was determined using the CycLex SIRT3 Deacetylase Fluorometric Assay Kit according to the manufacturer’s instructions (MBL International Corp. Japan).

### RNA interference of SIRT3

The siRNA for SIRT3 was purchased from Santa Cruz Biotechnology (Santa Cruz, CA, USA). Granulosa cells were transfected with 100 nM SIRT3 small interfering and non-targeted control siRNA according to the manufacturer’s protocol. Twenty-four hours after transfection, cells were harvested for further experiments.

### Chromatin immunoprecipitation (ChIP)

A ChIP assay was performed using the Pierce Agarose ChIP kit as described previously^23^. The relative binding of FoxO3a to the SOD2 and CAT promoters was determined using an ABI StepOnePlus PCR system (Applied Biosystems), SYBR Premix ExTaq II (TaKaRa) and the following primer sequences, SOD2: 5′-TTATGGAAACATTTGATAGCCACTGCTTCTTAGAC-3′ (Forward) and 5′-CGCGTGCTTGCTACAGCCACGC-3′ (Reverse); CAT: 5′-GCCAACAAGATTGCCTTCTC-3′ (Forward) and 5′-ACTGTCCGACATGGTGTAGGATT-3′ (Reverse). Techniques are described in more detail in Supplemental materials.

### Statistical analysis

Data (means ± SD) were analyzed using SPSS software (SPSS Inc., Chicago, IL, USA). Differences were analyzed using one-way analysis of variance (ANOVA) or *t*-test. For statistical analysis of the percentage values, the χ^2^ test were performed. Differences at P < 0.05 were considered to be statistically significant.

## Results

### Melatonin counteracted age-related fertility decline

Initially, we first determined plasma melatonin levels in female mice treated with melatonin and in the mice of the same age without treatment. Plasma melatonin levels in treated-mice significantly increased at both times tested in compared to the respective control group (Table 1). The values showed a peak of melatonin in the middle of the dark period, and the endogenous melatonin levels significantly decreased at the end of the dark period. However, in melatonin-treated mice, the plasma levels remained constantly higher compared with untreated mice (Supplementary Table S1).

Next, to determine whether supplementation with melatonin was able to rescue the reduced fertility associated with aging, mice treated with melatonin, which showed only a slightly increased litter size. In mice at the age of 14–16 mo, the average number of pups in the melatonin treated groups was significantly higher than from age-matched mice not receiving melatonin. It is notable that there was an increased pregnancy failure in the aged female mice following successful mating regardless of melatonin treatment, as indicated by the presence of mating plugs and mice bearing pups. These data indicate that the melatonin delays age-associated infertility, but could not completely prevent eventual reproductive aging (Table 2 and Supplementary Table S2).

### Melatonin attenuates the loss of follicles and follicle atresia

**Figure 1:**
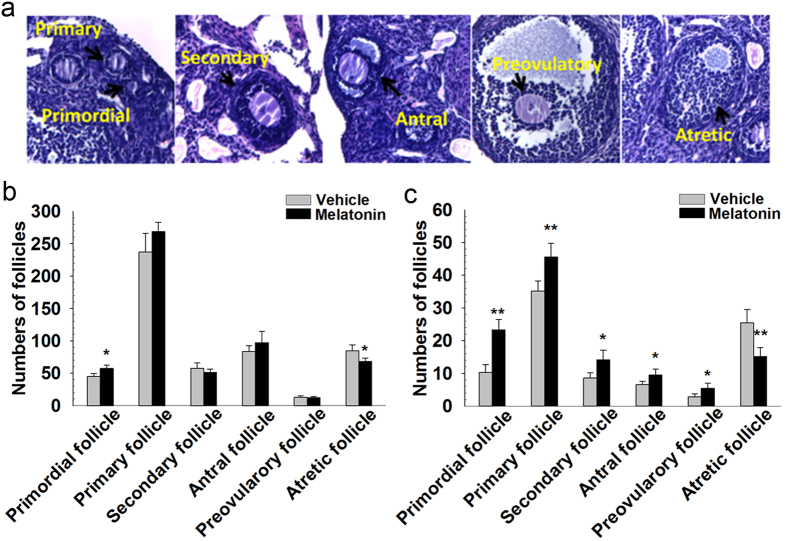
Follicle counts in mice treated with melatonin for 6 or 12 mo. (**a**) Follicles at different developmental stages in the ovaries. Numbers of different types of follicles in the ovaries of female mice exposed orally to melatonin for 6 (**b**) or 12 mo (**c**) Bars represent means ± SD (n = 5/group). *P < 0.05; **P < 0.01 versus the Vehicle group.

Follicles at different developmental stages in the ovaries were shown in Fig. 1a. Mice treated with melatonin for 6 mo had more primordial follicles compared with untreated age-matched mice. The number of atretic follicles was significantly decreased in aged mice with melatonin treatment. Melatonin administration for 6 mo did not, however, improve the number of growing and mature follicles (Fig. 1b). The numbers of primordial and primary follicles, and few growing and mature follicles continued to decline with age, but were higher in mice treated with melatonin for 12 mo than in age-matched untreated mice, the number of atretic follicles was significantly lower in mice treated with melatonin at this age (Fig. 1c).

### Melatonin increases the quantity and quality of oocytes

Next, we observed the oocytes retrieved from ovaries and oviducts of mice. As shown in Table 3 and Supplementary Table S3, the maternal age had a striking effect on the mean number of oocytes retrieved from ovaries and oviducts per mouse. The mean numbers of both ovarian GV oocytes and ovulated MII oocytes were higher in mice treated with melatonin for 12 mo than in age-matched untreated mice.

*In vitro* maturation of GV oocytes were shown in Table 4 and Supplementary Table S4. In mice at the age of 14–16 mo, maturation rate of oocytes from mice treated with melatonin was significantly lower than those of oocytes from the age-matched untreated mice.

Pronuclear formation of oocytes from mice of different ages in 4–7 h of IVF were shown in Table 5 and Supplementary Table S5. Pronuclear formation was accomplished in 7 h of IVF and the rate of oocytes forming pronuclei has no significant difference in each group. In mice at the age of 14–16 mo, the pronuclei formed in oocytes from aged mice without treatment later than that from mice treated with melatonin. Early embryonic development of oocytes after IVF is also shown in Table 5. Mice treated with melatonin for 12 mo increased the rates of oocytes developing to 2-cell and blastocyst than that of age-matched mice without treated.

**Figure 2:**
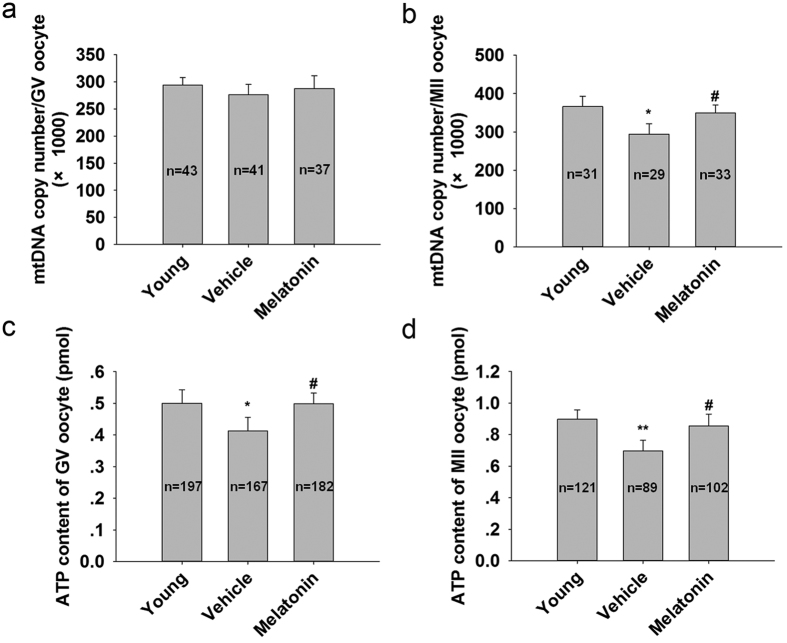
MtDNA copy numbers and ATP levels after ovulation induction following melatonin treatment for 12 mo. (**a**) MtDNA copy number per GV oocyte. (**b**): MtDNA copy number per MII oocyte. (**c**): ATP content per GV oocyte. (**d**) ATP content per MII oocyte. Data are means ± SD, n shows number of oocytes. *P < 0.05; **P < 0.01 versus the Young group. ^#^p < 0.05 versus the Vehicle group.

Reports indicate that insufficient mtDNA levels and ATP contents could be directly associated with diminished oocyte competence^24^. The data showed that mtDNA copy numbers in GV oocytes in the vehicle group showed no difference from that of mice treated with melatonin for 12 mo (Fig. 2a). However, the average mtDNA copy numbers in MII oocytes from melatonin-treated mice were significantly higher compared with the age-matched mice without treatment (Fig. 2b). Mice treated with melatonin for 12 mo significantly increased the ATP levels both in GV (Fig. 2c) and MII oocytes (Fig. 2d).

**Figure 3:**
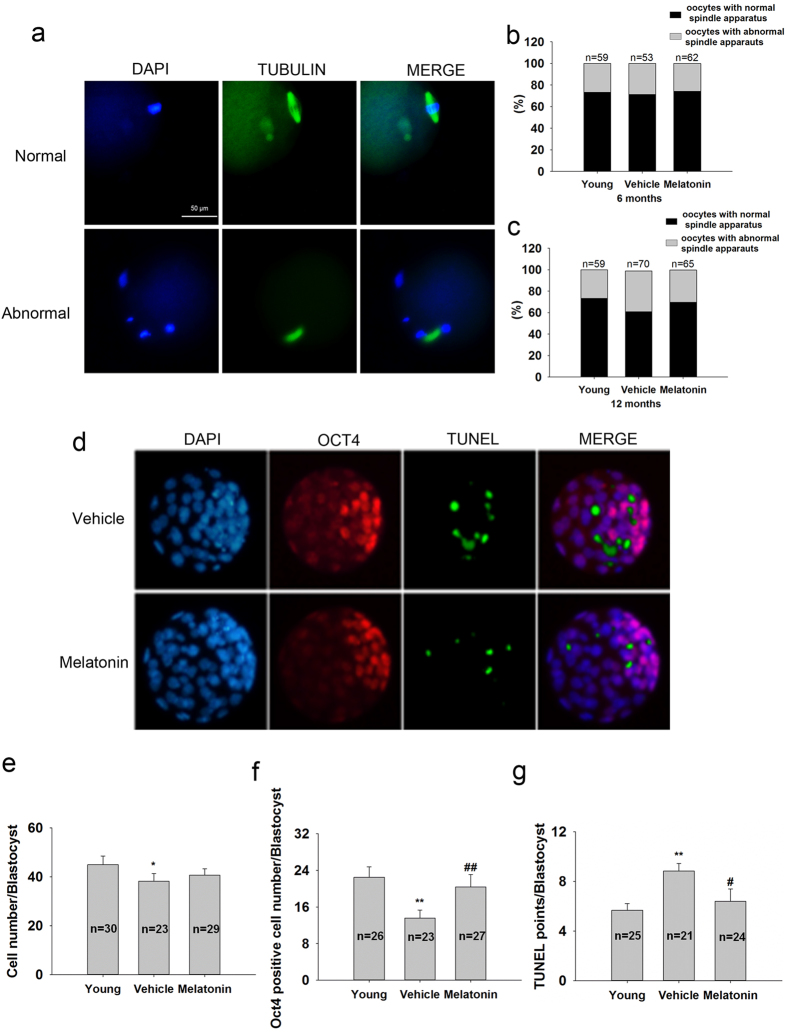
Oocyte quality and embryo development potential after ovulation induction following melatonin treatment. (**a**) Representative images of normal or abnormal chromosomes and spindle apparatus. A normal spindle apparatus was characterized as having chromosomes located exclusively on the equatorial plate and a barrel-shaped structure. Percentage of oocytes with normal or abnormal spindle apparatus following melatonin treatment for 6 (**b**) or 12 mo (**c**). (**d**) Representative immunofluorescence images for Oct4 (red) and TUNEL (green) staining in embryos cultured for 108 h. Nuclei stained with DAPI (blue). (**e**) Cell number in each embryo estimated by counting nuclei. (**f**) Number of Oct4-positive cells, indicative of inner cell mass cell number of each embryo. (**g**) Number of TUNEL-positive cells of each embryo. n = number of oocytes or embryos counted. The bars show means ± SD. *P < 0.05; **P < 0.01 versus the Young group. ^#^p < 0.05; ^##^p < 0.01 versus the Vehicle group.

Oocyte quality was further determined by spindle morphology and chromosome alignment (Fig. 3a). The frequency of normal oocytes in the untreated group showed no difference from that of mice treated with melatonin for 6 mo (Fig. 3b). Mice treated with melatonin for 12 mo exhibited a higher frequency of normal oocytes than age-matched untreated control (Fig. 3c). Oct4 immunostaining not only marks pluripotent cells but also indicates the inner cell mass of blastocysts^25^. Mice treated with melatonin for 12 mo did not influence the total number of cells in blastocysts (Fig. 3d,e), but significantly increased the number of Oct4-positive cells (Fig. 3d,f) and reduced the frequency of apoptosis (Fig. 3d,g) in blastocysts.

### Melatonin suppressed ovarian mitochondrial oxidative stress and apoptosis

**Figure 4:**
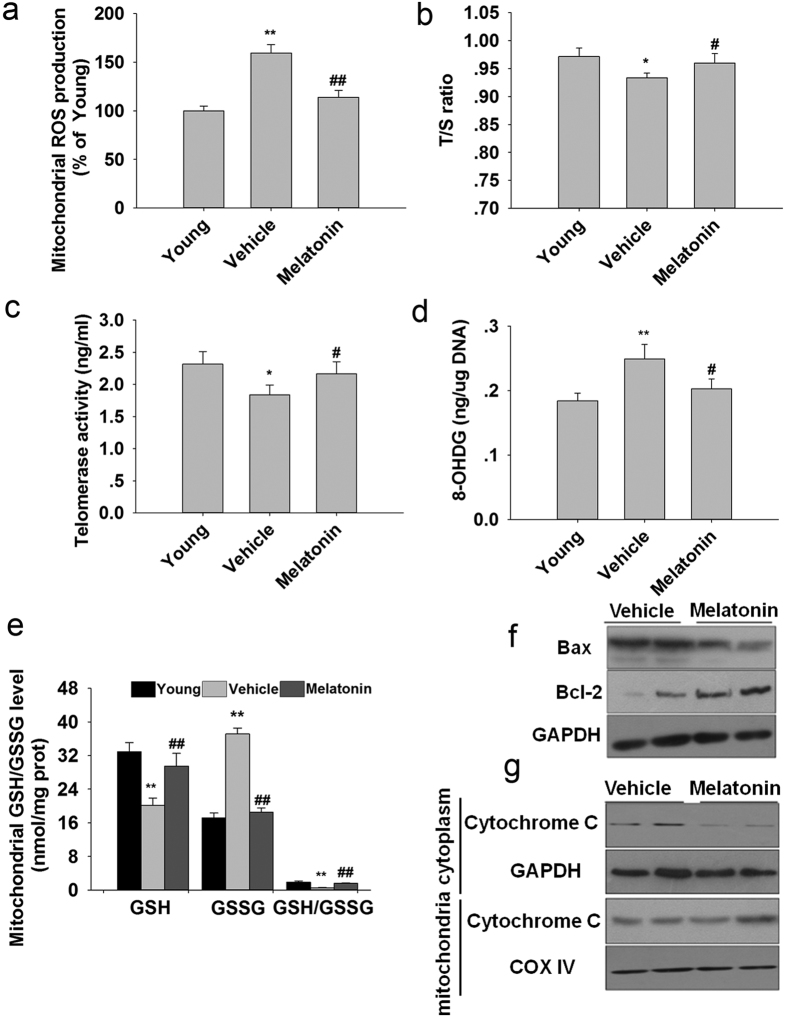
Mitochondrial oxidative stress and apoptosis in the ovaries of mice following melatonin treatment for 12 mo. (**a**): Mitochondrial ROS production. (**b**) The average telomere lengths of ovaries, shown as the T/S ratio, were determined by qRT-PCR. (**c**) Telomerase activities in the ovaries were analyzed by ELISA. (**d**) Indicators of mitochondrial DNA oxidation (8-OHdG). (**e**) GSSG/GSH ratio. Mitochondrial-related apoptosis were analyzed by western blotting. (**f**) Bax/Bcl-2 ratio. (**g**) Mitochondrial and cytosolic cytochrome c. Data are means ± SD (n = 7/group). *P < 0.05; **P < 0.01 versus the Young group. ^#^p < 0.05; ^##^p < 0.01 versus the Vehicle group.

Mice treated with melatonin significantly decreased mitochondrial ROS production (Fig. 4a). Telomeres erode rapidly in response to oxidative stress. Ovaries from mice treated with melatonin for 12 mo had longer telomeres than those from age-matched mice without treatment (Fig. 4b). Telomerase activity in mice treated with melatonin for 12 mo was higher than in age-matched untreated mice (Fig. 4c). Aged mice treated with melatonin for 12 mo had markedly reduced 8-OHdG level in mitochondria compared with those of age-matched untreated mice (Fig. 4d). Long-term treatment with melatonin prevented aging-associated oxidative stress in the ovarian mitochondria, as assessed by the recovery of GSH levels and GSH/GSSG ratios (Fig. 4e).

Melatonin treatment significantly suppressed Bax protein expression and decreased the Bax/Bcl-2 ratio (Fig. 4f) and decreased cytoplasmic cytochrome c levels (Fig. 4g) compared with age-matched mice without treatment.

### Melatonin attenuated ovarian mitochondrial dysfunction

**Figure 5:**
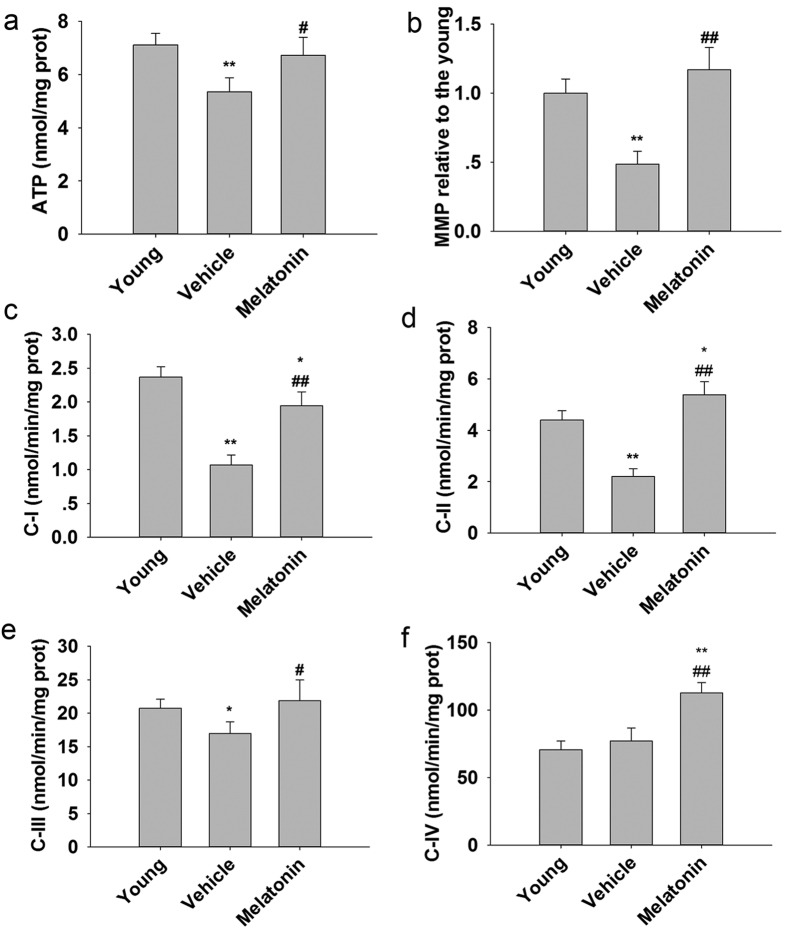
Mitochondrial function in the ovaries of mice following administration of melatonin for 12 mo. (**a**) ATP level. (**b**) MMP value. (**c**) Mitochondrial complex I activity. (**d**) Mitochondrial complex II activity. (**e**) Mitochondrial complex III activity. (**f**) Mitochondrial complex IV activity. Data are means ± SD (n = 10/group). *P < 0.05; **P < 0.01 versus the Young group. ^#^p < 0.05; ^##^p < 0.01 versus the Vehicle group.

In the ovarian mitochondria from female mice treated with melatonin for 12 mo, ATP content (Fig. 5a) and MMP (Fig. 5b) were significantly higher than in age-matched untreated females. Mitochondria, especially mitochondrial respiratory chain, has been shown to be major sources of ROS^26^. We measured the activities of complexes I-IV of mitochondrial electron transport chain (ETC). Aging did not affect activity of complex IV (Fig. 5f), but there was a significant decrease in activities of complex I (Fig. 5c), II (Fig. 5d) and III (Fig. 5e) in untreated mice at 14–16 mo of age compared with in young mice. Administration of melatonin resulted in a remarkable augmentation of the ETC activities, as compared with age-matched mice without treatment. In agreement with the reduced mitochondrial ROS production, long-term treatment with melatonin significantly elevated the ETC activities.

### Melatonin protects granulosa cells from oxidative stress *in vitro*

**Figure 6:**
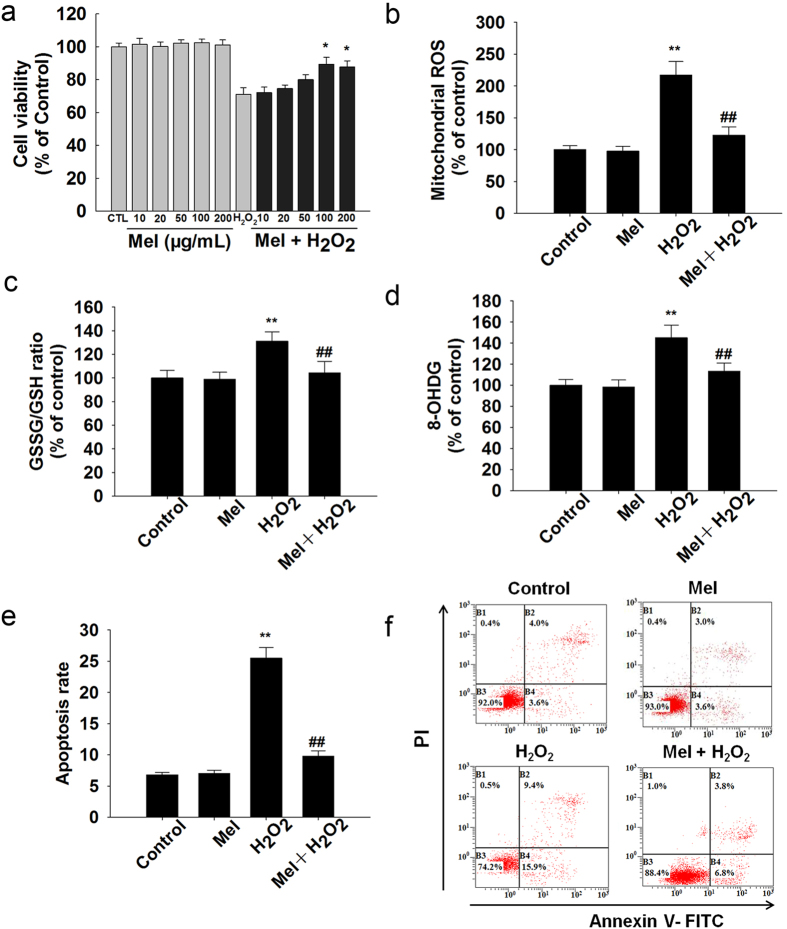
Effect of melatonin on the H_2_O_2_-induced oxidative damage and apoptosis in granulosa cells *in vitro*. (**a**) Cell viability was measured by CCK-8. (**b**) Mitochondrial reactive oxygen species (mROS). (**c**) GSSG/GSH ratio. (**d**) The elevation of mitochondrial oxidative stress was further confirmed by 8-OHdG. (**e**,**f**): Apoptosis rate. All values are the mean ± SD of the results from three independent experiments. **p < 0.01 versus the Control group. ^#^p < 0.05; ^##^p < 0.01 versus the H_2_O_2_ group.

Granulosa cell is one of the main components of ovary and it is a appropriate object for *in vitro* experiments. According to lots of previous studies, granulosa cells were examined to indicate ovarian aging in the *in vitro* experiments. The H_2_O_2_-induced decrease in cell viability was significantly attenuated after treatment with melatonin (Fig. 6a). *In vitro* analysis showed that melatonin blocked mitochondrial ROS elevation induced by H_2_O_2_ in granulosa cells (Fig. 6b). Moreover, co-incubation with melatonin prevented H_2_O_2_-induced oxidative stress (Fig. 6c), DNA damage (Fig. 6d) and apoptosis (Fig. 6e,f).

### Melatonin upregulates SOD2 and CAT through the interaction of SIRT3 with FoxO3a *in vitro*

We firstly examined whether the expression of aging-related SIRT3 is modulated by oxidative stress in granulosa cells. Interestingly, the results revealed that granulosa cells stressed with H_2_O_2_ induced a significant increase in SIRT3 expression (Supplementary Fig. S1a) and activity (Supplementary Fig. S1b), however, it dropped significantly after two hours. Cell viability was further determined using the CCK-8 assay (Supplementary Fig. S1c). Overexpression of SIRT3 restore the cell viability (Supplementary Fig. S1e), while SIRT3-siRNA exhibited a marked reduction in cell viability (Supplementary Fig. S1d) in response to H_2_O_2_, indicating that SIRT3 is necessary to prevent granulosa cells against senescence-related oxidative stress.

**Figure 7:**
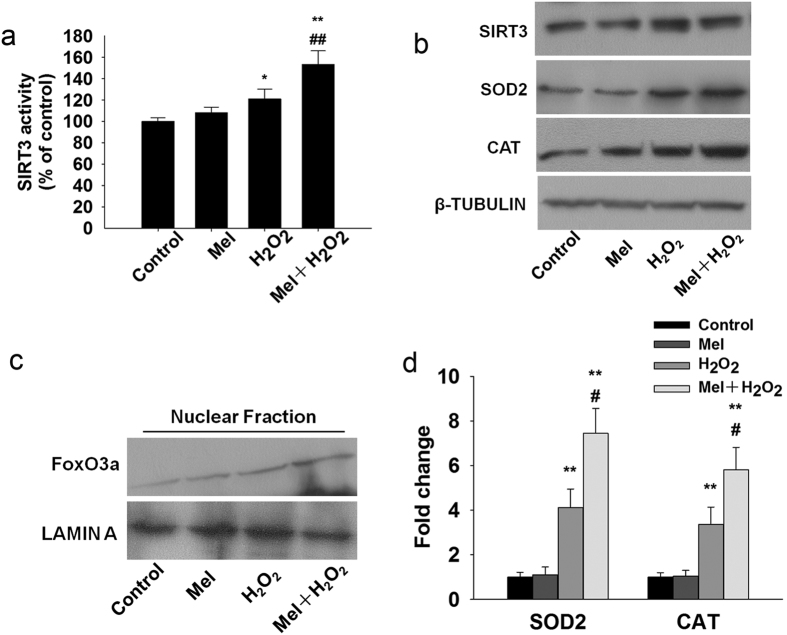
Melatonin increase the SIRT3 activity, nuclear translocation of FoxO3a and binding of FoxO3a to the promoters of SOD2 and CAT in granulosa cells following H_2_O_2_ treatment. (**a**) Graph shows a significant increase in the SIRT3 activity. (**b**) Western blots showed the expression of SIRT3, SOD2 and CAT in granulosa cells. (**c**) Western blot analysis shows the nuclear translocation of FoxO3a in granulosa cells. (**d**) FoxO3a has a strong binding affinity to the promoters of mitochondrial antioxidants SOD2 and CAT in granulosa cells. *p < 0.05; **p < 0.01 versus the Control group. ^#^p < 0.05; ^##^p < 0.01 versus the H_2_O_2_ group. All values are the mean ± SD of the results from three independent experiments.

Our data showed that melatonin enhanced the SIRT3 activity (Fig. 7a) without significantly affecting SIRT3 protein levels (Fig. 7b) in response to oxidative stress. FoxO3a protein expression significantly increased in the nuclear fraction of granulosa cells treated with melatonin compared to control (Fig. 7c) under oxidative stress. In ChIP assay, we found that SIRT3 promoted the binding of FoxO3a to the promoter of SOD2 and CAT (Fig. 7d). The increased protein expression of SOD2 and CAT were further confirmed with western blot analysis.

**Figure 8:**
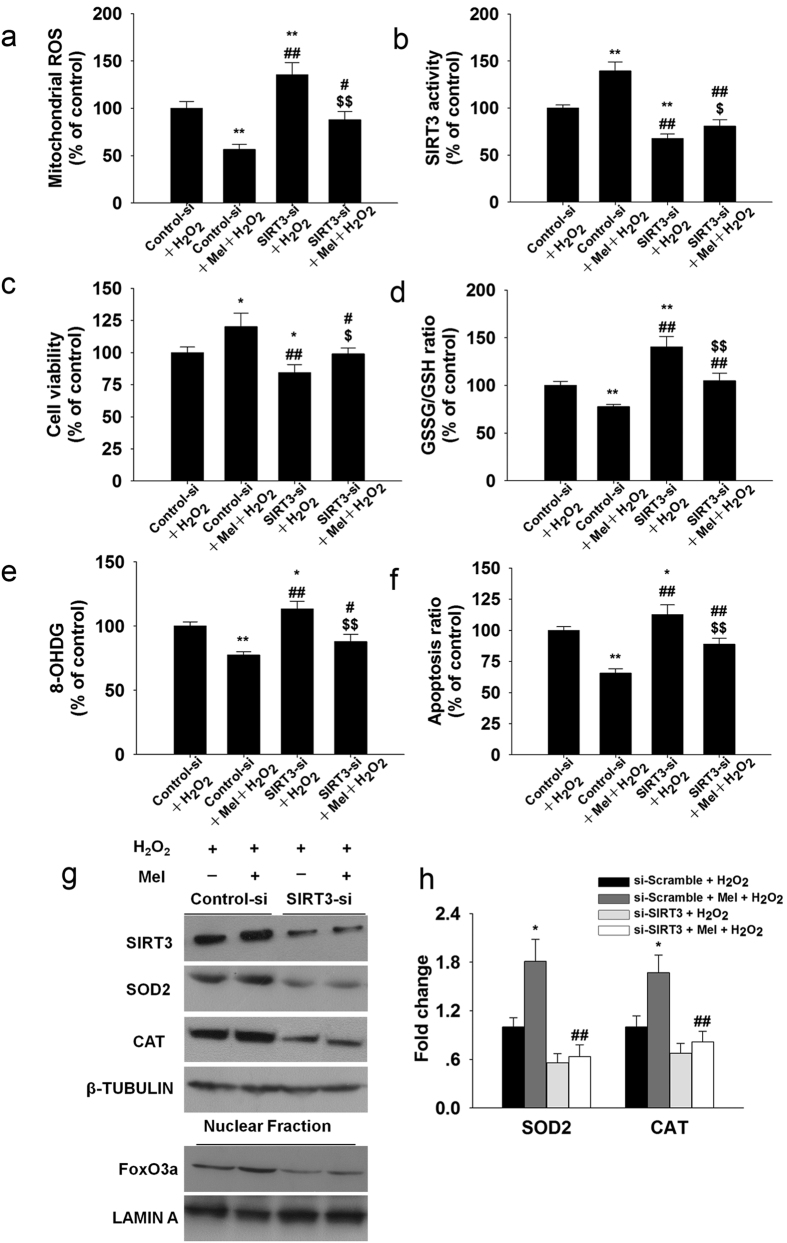
SiRNA-mediated knockdown of SIRT3 abolishes the protective effects of melatonin in granulosa cells. SIRT3 was knocked down by SIRT3 siRNA transfection as described in the Materials and Methods section. (**a**) Mitochondrial ROS, (**b**) SIRT3 activity, (**c**) Cell viability, (**d**) GSSG/GSH ratio, (**e**) 8-OHdG, (**f**) Apoptosis ratio. (**g**) The expressions of SIRT3, SOD2, CAT and the nuclear translocation of FoxO3a were examined using immunoblotting. (h) Granulosa cells were harvested for ChIP analysis and the FoxO3a binding affinity was measured by real-time PCR assay. The values are presented as the mean ± SD of the results from three independent experiments. *p < 0.05, **p < 0.01 versus the Control siRNA + H_2_O_2_ group; ^#^p < 0.05, ^##^p < 0.01 versus the Control siRNA + H_2_O_2_ + melatonin group; ^$^p < 0.05, ^$$^p < 0.01 versus the SIRT3 siRNA + H_2_O_2_ group. For ChIP assay, *p < 0.05 versus the si-Scramble + H_2_O_2_ group; ^##^p < 0.01 versus the si-Scramble + Mel + H_2_O_2_ group.

SIRT3 siRNA treatment significantly attenuated the protective effects of melatonin on H_2_O_2_-induced mitochondrial ROS generation (Fig. 8a) and cell viability (Fig. 8c). Furthermore, GSSG content (Fig. 8d), 8-OHdG (Fig. 8e) and apoptosis (Fig. 8f) were decreased in cells with melatonin, but these beneficial effects were abolished in the presence of SIRT3 siRNA. SIRT3 activity (Fig. 8b) and protein level (Fig. 8g) and FoxO3a nuclear localization (Fig. 8g) were significantly reduced in granulosa cells subjected to siRNA-mediated SIRT3 knockdown. Upon SIRT3 knockdown by siRNA, SOD2 and CAT levels were significantly downregulated in granulosa cells (Fig. 8g), and the binding affinity of FoxO3a to SOD2 and CAT promoters were significantly decreased (Fig. 8h).

### Melatonin increased the SIRT3 and FoxO3a expression *in vivo*

**Figure 9:**
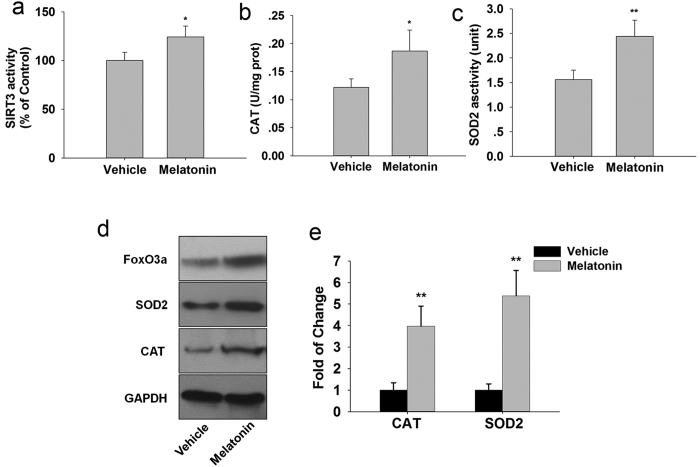
Melatonin suppresses ovarian mitochondrial oxidative stress by enhancing SIRT3 activity *in vivo*. (**a**) The effects of melatonin on SIRT3 activity. Mitochondrial antioxidants activities of CAT (**b**) and SOD2 (**c**) were measured. (**d**) Western blot were further confirmed the expression of FoxO3a, SOD2 and CAT. (**e**): The binding of FoxO3a to the promoters of SOD2 and CAT were performed by ChIP-qPCR in ovary. Data are presented as the mean ± SD (n = 7/group). *p < 0.05, **p < 0.01 versus the Vehicle group.

Mice receiving melatonin had significantly higher activities of SIRT3 (Fig. 9a), CAT (Fig. 9b) and SOD2 (Fig. 9c) in the ovaries than did the untreated aged mice. We further investigated the role of FoxO3a in melatonin-induced CAT and SOD2 expression (Fig. 9d) using chromatin immunoprecipitation. In ChIP assay, we found that melatonin promoted the binding of FoxO3a to the promoters of CAT and SOD2 (Fig. 9e).

## Discussion

In the present study we show that exogenous melatonin on the protects against the reduction during the aging of mice as indicated by litter size. Furthermore, melatonin improves the healthy follicle number, telomerase activity and telomere length, as well as the oocyte quality and quantity, indicating that it staves off the ovarian aging.

The well-established paradigm of reproduction in mammals holds that females are born with a fixed number of oocytes which continuously decline until few or none remain^27^. Our results are in agreement with studies, that aging ovaries exhibit reduced numbers, as well as reduced quantity and quality of oocytes. We show that melatonin may provide long-term beneficial effect by maintaining female reproductive capacity in mice. The beneficial effects of melatonin on ovarian aging might be contribute to the slowed loss of non-renewable female germ cells. But, we should note that melatonin rescue experiments showed that melatonin cannot prevent but can significantly delay declined fertility associated with reproductive age.

It has been suspected that the reproductive failure can be attributed to the functional and structural qualities of the oocytes^28^. Reports indicate that sub-normal mtDNA levels and ATP contents could be directly associated with poor oocyte quality and detrimental embryonic development^29^. It seems that the reduced number of oocytes retrieved with age may be of less importance than the decline in oocyte quality. Age-related fertility decline of the mice is not only due to a reduced oocyte pool, but also the result of a decrease in oocyte quality and embryonic development potential. Melatonin treatment both improves the quantity and quality of oocytes with age.

Various theories propose an essential role of mitochondria in cellular events associated with the aging process, via accumulation of mitochondrial ROS and oxidative damage to mitochondrial and cytoplasmic components^30^,^31^. There is evidence that mitochondrial respiratory complex activity and mitochondrial membrane potential decline during aging and that endogenous antioxidant system activities exhibit an age-dependent decrease, making redox imbalance play a more predominant role in the aged cells and tissues. Here we showed that melatonin was effective in ameliorating mitochondrial oxidative damage, apoptosis and preserving mitochondrial function in the aged ovarian. There is increasing evidence for beneficial effects of the antioxidant melatonin in preventing ROS-induced damage and pathology^32^,^33^. Reducing oxidative stress by supplementation of melatonin could potentially reduce mitochondrial ROS-induced damage, thus maintaining the number and quality of oocytes and follicles. Telomere shortening is considered as a biomarker of cellular senescence and is highly sensitive to oxidative stress^34^,^35^. Telomere dysfunction may contribute to reproductive aging-associated meiotic defects, miscarriage and infertility^36^. Thus, protection against oxidative stress by melatonin (Supplementary Fig. S2) could directly contribute to maintenance of telomeres in aging mouse ovaries.

As one of the main components of ovary, granulosa cell is a appropriate object for *in vitro* experiments. Ovarian ageing involves the accumulation of damage by oxidative stress associated with an increasingly compromised microcirculation around the leading follicle^37^. Previous study have established that advanced maternal age have altered expression of genes involved in mitochondrial metabolic pathways in granulosa and cumulus cells compared with young^38^,^39^. Communication between the oocyte and its surrounding cells is essential for the development of a viable oocyte, and impaired mitochondria (Supplementary Fig. S3) within the follicular cells ultimately affect oocyte development. Moreover, follicular atresia is the main process responsible for the loss of follicles and oocytes from the ovary, and it is the root cause of ovarian aging. More than 99% of follicles undergo atresia that has been associated with apoptosis of granulosa cells^40^. Therefore primary ovarian granular cells were further selected for *in vitro* experimentation.

SIRT3 is the primary mitochondrial deacetylase, and it regulates biological functions directly involved in mitochondrial function and limits the generation of mitochondrial ROS^41^. Residing predominantly in the mitochondria, SIRT3 is known to deacetylate and activate complex I of the respiratory chain in mitochondria, leading to an increase in levels of ATP and thereby protecting the cells from ROS-mediated oxidative damage^42^. Ectopic SIRT3 expression has antioxidant regulatory effects in primary cardiomyocytes, suggesting that SIRT3 may regulate antioxidant systems^43^. The activity of sirtuin 3 (SIRT3) was decreased in granulosa and cumulus cells from women of advanced maternal age^44^. SIRT3 attenuation or ablation is associated with accelerated development of several diseases of aging^45^.

SIRT3 has also been shown to interact with FoxO3a, the transcription factor implicated in ROS regulation and to activate FoxO3a-dependent gene expression^46^,^47^. Increased nuclear translocation of FoxO3a strengthens antioxidant defense systems, which leads to extended longevity, in several experimental organisms^48^,^49^. An age-mediated decrease in FoxO3a activity was observed in aged rats, which led to a decrease in the mitochondrial antioxidant enzymes^50^. In this study as FoxO3a was found to bind to the promoter regions of SOD2 and CAT genes. These results indicate that melatonin enhances the SIRT3 activity and activates FoxO3a nuclear translocation which leads to transactivation of the anti-oxidant genes, thereby resulting in inhibition of mitochondrial oxidative damage. Notably, though melatonin could scavenge the H_2_O_2_ directly, H_2_O_2_ radical scavenging ability of melatonin works mainly through the SIRT3 pathway (Supplementary Fig. S4). Overall, SIRT3 and its target FoxO3a appear to function against mitochondrial oxidative damage.

In mammals, overall reproductive lifespan is predetermined at birth by the number of oocytes in the ovary and the ovarian aging manifestations of menopause occur when the finite supply of oocytes is depleted. However, our results using mice as a model suggest that this depletion can be significantly delayed, with potential clinical benefits for women who wish to postpone childbearing. More extensive research will be needed, however, prior to conducting clinical trials to test this proposal.

## Additional Information

**How to cite this article**: Song, C. *et al.* Melatonin improves age-induced fertility decline and attenuates ovarian mitochondrial oxidative stress in mice. *Sci. Rep.* **6**, 35165; doi: 10.1038/srep35165 (2016).

## Supplementary Material

Supplementary Information

## Figures and Tables

**Table 1:** Melatonin levels in plasma of treated and untreated female mice.

Length of treatment (mo)	Group	Melatonin Levels (pg/mL)
6	Vehicle	26.2 ± 3.7
	Melatonin	43.0 ± 2.1**
12	Vehicle	21.8 ± 5.3
	Melatonin	75.5 ± 6.8**

Serum melatonin levels at different times were determined. Un-paired Student’s t-test was used to compare two groups. **p < 0.01.

**Table 2:** Effect of melatonin on female fertility.

Length of treatment (mo)	Groups	No. of mice examined	No. of mice with vaginal plug	No. of mice bearing pups	Total number	Average litter size of pregnant mice
6	Vehicle	23	19	17	109	6.5 ± 2.3
	Melatonin	21	20	20	162	7.1 ± 1.8
12	Vehicle	29	22	11	32	2.9 ± 1.6
	Melatonin	25	21	16	90	5.7 ± 2.2**

*P < 0.05, **P < 0.05 versus vehicle; values are mean ± SD.

**Table 3:** Effect of melatonin on number of oocytes retrieved from ovaries and oviducts.

Length of treatment (months)	Groups	No. of mice examined	Ovarian GV oocytes		No. of mice examined	Ovulated MII oocytes	
			Total number	Number of oocytes recovered/mouse Mean ± SD (n)		Total number	Number of oocytes recovered/mouse Mean ± SD (n)
6	Vehicle	13	402	30.5 ± 5.7	12	227	18.2 ± 4.4
	Melatonin	12	386	31.9 ± 4.5	12	239	19.7. ± 5.6
12	Vehicle	19	313	16.3 ± 4.1	26	118	4.5 ± 3.8
	Melatonin	19	447	23.2 ± 3.2*	19	186	9.6 ± 3.4*

*P < 0.05, **P < 0.01 versus vehicle; values are mean ± SD.

**Table 4:** Effect of melatonin on *in vitro* maturation of GV oocytes.

Length of treatment (mo)	Groups	No. of mice examined	No. of oocytes cultured	Maturation stage examined in 16–18 h of culture (%)		
				GV	MI	MII
6	Vehicle	13	395	34 (8.6)	21 (5.3)	340 (86.1)
	Melatonin	13	404	32 (7.9)	24 (6.1)	350 (86.6)
12	Vehicle	21	366	41 (11.2)	33 (9.0)	301 (79.8)
	Melatonin	17	390	36 (9.7)	27 (6.9)*	339 (83.4)*

*P < 0.05, **P < 0.01 versus vehicle; χ^2^ test was used.

**Table 5:** Effect of melatonin on pronuclear formation and development of MII oocytes after *in vitro* fertilization.

Length of treatment (mo)	Groups	No. of mice examined	No. of oocytes cultured	No. of oocytes forming pronuclei (%)				No. of embryo (%)	
				4 h	5 h	6 h	7 h	2-cell	Blastocysts
6	Vehicle	12	227	15(6.6)[7.4]	76(33.5)[37.6]	187(82.4)[92.6]	202(89.0)[100]	181(79.8)	139(61.2)
	Melatonin	12	235	16(6.8)[7.7]	89(37.9)[42.6]	197(83.8)[94.3]	209(88.9)[100]	190(80.9)	149(63.4)
12	Vehicle	26	144	6(4.2)[5.1]	51(35.4)[43.2]	105(72.9)[89.0]	118(81.9)[100]	107(74.3)	80(55.6)
	Melatonin	19	186	21(11.3)*[13.5]	94(50.5)**[60.3]	150(80.6)*[96.2]	156(83.9)[100]	146(78.5)*	117 (62.9)*

Values in [] are represented the percentages of oocytes forming pronuclei at certain time of IVF/the total oocytes forming pronuclei.

*P < 0.05, **P < 0.01 versus vehicle; values are mean ± SD, by χ^2^ test.
