# Supplementary material for: Melatonin improves age-induced fertility decline and attenuates ovarian mitochondrial oxidative stress in mice
Source: Sci Rep. 2016 Oct 12;6:35165. doi: 10.1038/srep35165 (PMC5059725; doi:10.1038/srep35165)
Supplement: Supplementary Information [file srep35165-s1.doc]

**Supplementary Information**

**Title:** Melatonin improves age-induced fertility decline and attenuates ovarian mitochondrial oxidative stress in mice

Chao Song^a, b^, Wei Peng^a, b^, Songna Yin^a, b^, Jiamin Zhao^a, b^, Beibei Fu^a, b^, Jingcheng Zhang^a, b^, Tingchao Mao^a,b^, Haibo Wu ^a, b^,*, Yong Zhang ^a, b^,*

^a^College of Veterinary Medicine, Northwest A&F University, Yangling 712100, Shaanxi, China

^b^Key Laboratory of Animal Biotechnology, Ministry of Agriculture, Northwest A&F University, Yangling 712100, Shaanxi, China

*Correspondence should be addressed to Yong Zhang or Haibo Wu

Tel.: +86 29 87080092

Fax: +86 29 87080092

E-mail: zhangy1956@sina.com (Y.Z) or hbwu029@nwsuaf.edu.cn (H.W)

**Materials and methods**

**Oocyte collection**

To collect germinal vesicle (GV) stage oocytes, mice were primed with 10 IU pregnant mare’s serum gonadotropin (PMSG) (Ningbo Hormone Products Co., Ningbo, Zhejiang, People’s Republic of China) by intraperitoneal injection. After 48 h, cumulus enclosed oocytes were obtained by manual rupture of antral ovarian follicles. Oocytes were arrested at the GV stage in M2 medium by addition of 2.5 μM milrinone. To obtain ovulated oocytes, females were injected with 10 IU human chorionic gonadotropin (hCG) 48 h after PMSG priming. MII stage oocytes were recovered from oviductal ampullae 15 h after hCG following exposure to 1 mg/mL hyaluronidase to remove the cumulus cells. Fragmented or degenerated cells were discarded and only healthy oocytes were used for further research.

***In vitro* maturation (IVM) of oocytes, *in vitro* fertilization (IVF) and embryo culture**

GV oocytes were collected and cultured at 37 °C in a humid air atmosphere with 5 % CO_2_. *In vivo* matured MII oocytes were fertilized *in vitro*. Embryos were cultured to the blastocyst stage for 96 h for further analysis and the development rate was recorded every 24 h.

**Mitochondrial ROS production assay**

Mitochondrial samples or negative controls were incubated in a total volume of 200 μL respiration buffer (5 mM pyruvate and 2.5 mM malate) in the presence of CM-H_2_DCFDA (5 μM) at 37 °C for 25 min, with the dye solution freshly prepared before use. The relative amount of reactive oxygen species (ROS) production was measured at excitation and emission wavelengths of 488 and 530 nm, respectively.

**Plasmid construction and cell transfection**

SIRT3 whole length cDNA was synthesized using RT-PCR with total RNA extracted from granulosa cells as template and inserted into the SalI/XhoI site of the PCMV-HA expression vector (Clontech) with the following primers: SIRT3-F, 5' -ATAGTCGACCAGTAGGGTGGTGGTCAT- 3', and SIRT3-R, 5'- CATCTCGAGCCAGGTGAAGAAGCCATA- 3'. Cells were transfected using Opti-MEM reduced serum media and Lipofectamine 2000 according to the manufacturer’s instructions (Invitrogen).

**Chromatin immunoprecipitation (ChIP)**

The concrete steps for ChIP assay using the tissue of ovary are described as follows: Step 1. Chop fresh ovary into small pieces (1-3 mm3) with a scalpel. Transfer them into a 50 mL-tube with a screw cap lid and add a small (10 mL) amount of 1X PBS. Add formaldehyde to a final concentration of 1 % and rotate tube at room temperature for 15 minutes. Step 2. Stop the cross linking reaction by adding glycine to a final concentration of 0.125 M. Continue to rotate at room temp for 5 minutes. Step 3. Centrifuge samples at low speed (100 g) for 5 min at 4°C, decant supernatant and wash twice with cold 1X PBS. Centrifuge. Add a small (1-2mls per sample) amount of cold 1X PBS and disaggregate tissues. Centrifuge at low speed (1000 rpm/min) for 5 min at 4°C to pellet cells and decant supernatant. Step 4. Resuspend cell pellet in cell lysis buffer (provided by the kit) plus the protease inhibitors PMSF (10 μL per mL), leupeptin (1 μL per mL) and aprotinin (1μL per mL). Note: The final volume of cell lysis buffer should be sufficient so that there are no clumps of cells. Incubate on ice for 15 minutes. Step 5. Microfuge (5,000 rpm) for 5 minutes at 4°C to collect the pellet. Resuspend the cell pellet in nuclei lysis buffer plus the same protease inhibitors as the cell lysis buffer. Incubate on ice for 20 minutes, and then ultrasonication. Genomic DNA was isolated and sheared to average lengths of 300–500 bp by ultrasonic waves and 10 % of the supernatant was regarded as input. An antibody against FoxO3a was used for ChIP.

**Results**

**Supplementary Table S1** Melatonin levels in plasma of treated and untreated young female mice

| Time | Group | Melatonin Levels (pg/mL) |
| --- | --- | --- |
| 12 AM | Vehicle | 165.46 ± 5.82 |
|  | Melatonin | 226.19 ± 11.67^**^ |
| 4 AM | Vehicle | 104.55 ± 4.21 |
|  | Melatonin | 123.60 ± 7.45^*^ |
| 6 AM | Vehicle | 76.48 ± 6.73 |
|  | Melatonin | 111.31 ± 8.04^**^ |
| 9 AM | Vehicle | 41.73 ± 4.81 |
|  | Melatonin | 56.04 ± 5.16^*^ |

Melatonin levels in plasma of treated and untreated of young female mice were measured at different times. Un-paired Student’s t-test was used to compare two groups. *p<0.05, **p<0.01.

**Supplementary Table S2** Female fertility in young mice at the age of 2–3 mo

| Group | No. of mice examined | No. of mice with vaginal plug | No. of mice bearing pups | Total number | Average litter size of pregnant mice |
| --- | --- | --- | --- | --- | --- |
| Young | 9 | 9 | 9 | 128 | 14.2 ± 1.3 |

Young: Female mice at the age of 2–3 mo.

**Supplementary Table S3** Oocytes retrieved from ovaries and oviducts in young mice at the age of 2–3 mo

|  |  | Ovarian GV oocytes | |  | Ovulated MII oocytes | |
| --- | --- | --- | --- | --- | --- | --- |
| Group | No. of mice examined | Total number | Number of oocytes recovered/mouse Mean ± SD (n) | No. of mice examined | Total number | Number of oocytes recovered/mouse Mean ± SD (n) |
| Young | 14 | 823 | 62.2 ± 9.5 | 10 | 412 | 40.8 ± 11.5 |

Young: Female mice at the age of 2–3 mo.

**Supplementary Table S4** *In vitro* maturation of GV oocytes from young mice at the age of 2–3 mo

|  |  |  | Maturation stage examined in 16–18 h of culture (%) | | |
| --- | --- | --- | --- | --- | --- |
| Group | No. of mice examined | No. of oocytes cultured | GV | MI | MII |
| Young | 10 | 573 | 89 (15.6) | 61 (10.7) | 423 (73.8) |

Young: Female mice at the age of 2–3 mo.

**Supplementary Table S5** Pronuclear formation and development of MII oocytes after *in vitro* fertilization in young mice at the age of 2–3 mo

| Group | No. of mice examined | No. of oocytes cultured | No. of oocytes forming pronuclei (%) | | | |  | No. of embryo (%) | |
| --- | --- | --- | --- | --- | --- | --- | --- | --- | --- |
|  |  |  | 4 h | 5 h | 6 h | 7 h |  | 2-cell | Blastocysts |
| Young | 8 | 312 | 174 (55.9)[63.0] | 250 (78.4)[90.6] | 266 (85.3)[96.4] | 276 (88.5)[100] |  | 251(80.4) | 215(68.8) |

Values in [] are represented the percentages of oocytes forming pronuclei at certain time of IVF/the total oocytes forming pronuclei.


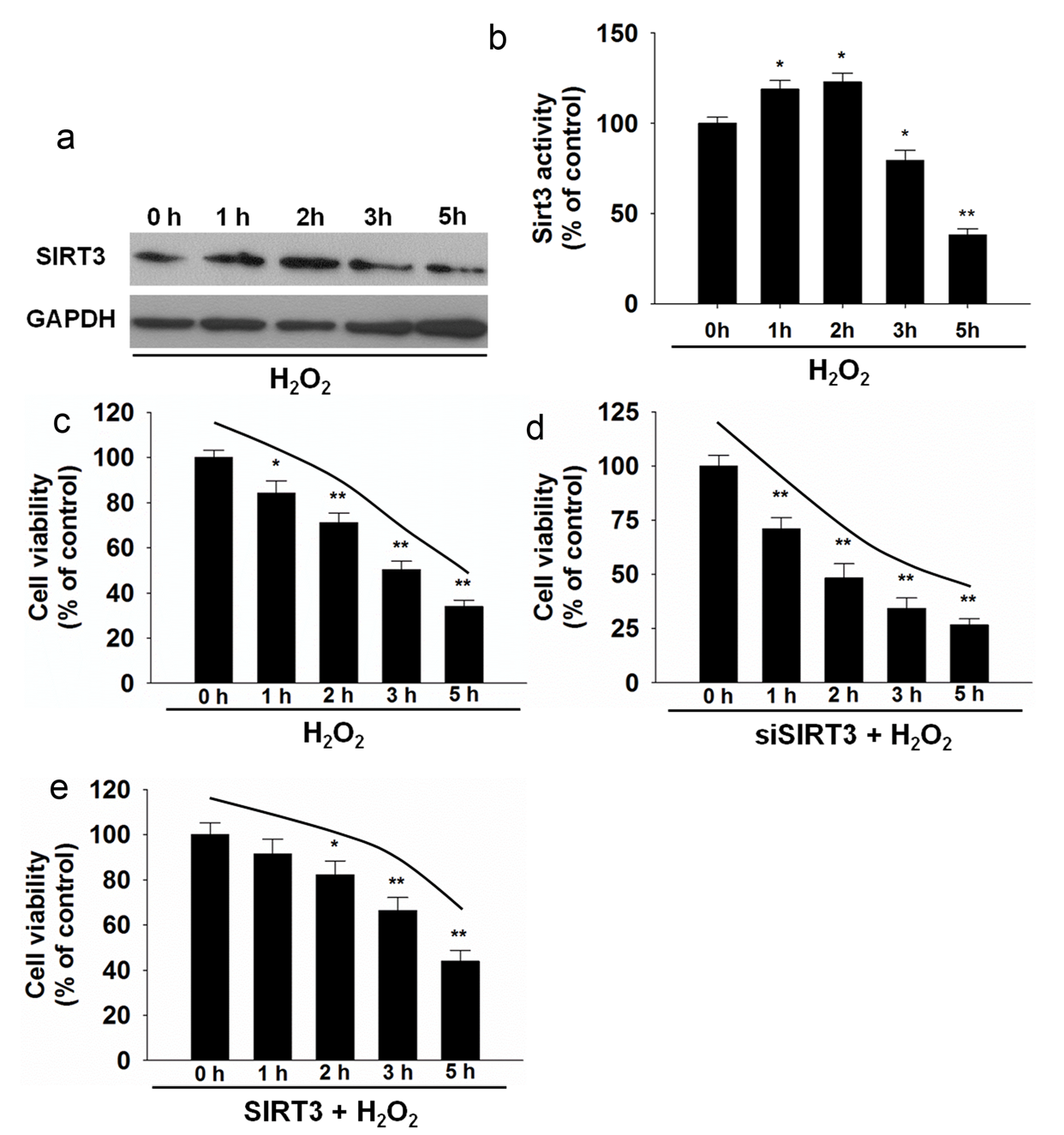


**Supplementary Figure S1. SIRT3 expression and activity in granulosa cells under H_2_O_2_ exposure.** Cells were treated with 2 mM H_2_O_2_ for different times (0, 1, 2, 3 and 5 h). (a): SIRT3 expression, (b): SIRT3 activity, (c): Cell viability, (d): SIRT3 siRNA treatment promoted cell death. (e): Overexpressed SIRT3 decreased H_2_O_2_-induced cell death. All values are the mean ± SD of the results from three independent experiments. ^*^p < 0.05; ^**^p<0.01 versus the 0 h, respectively.


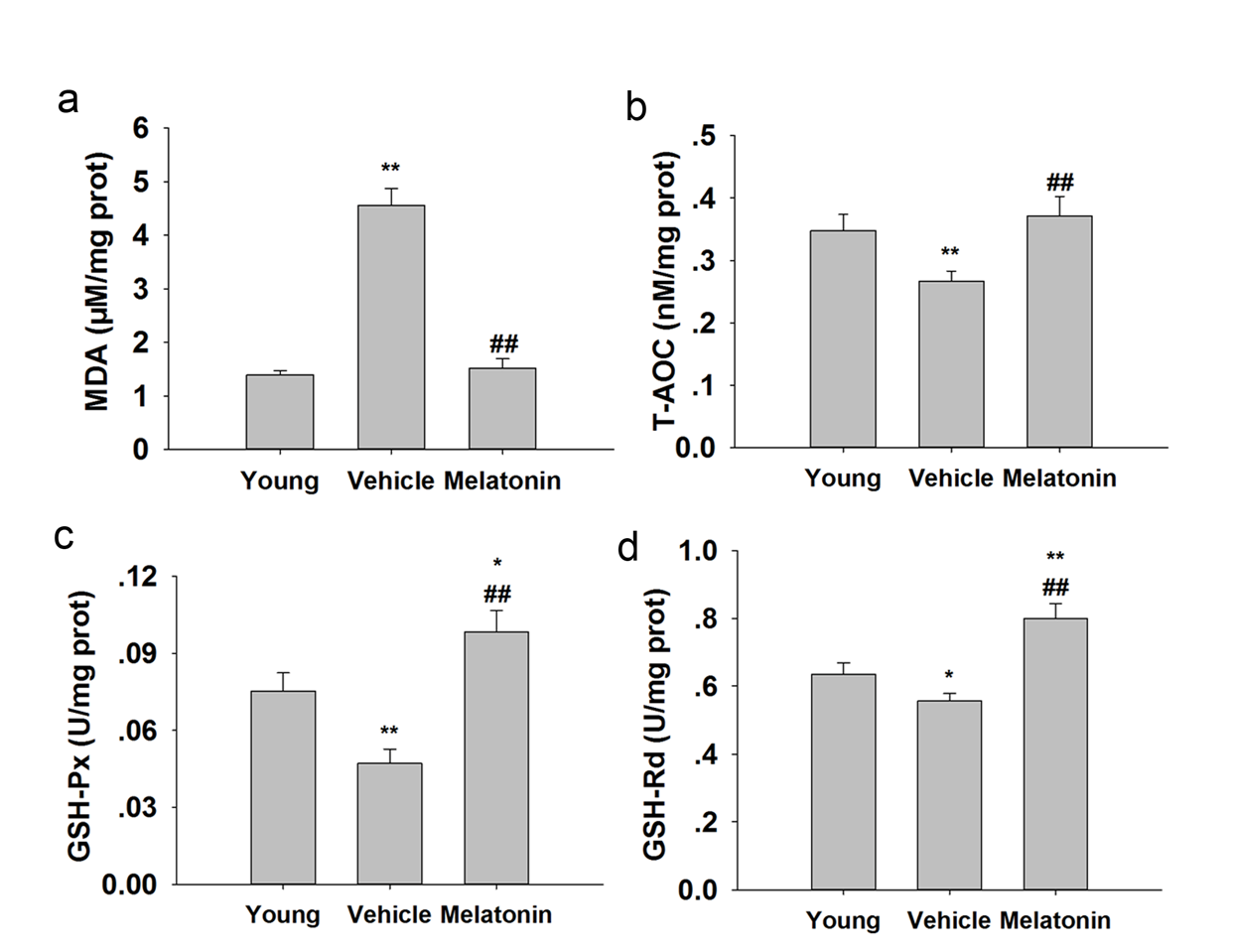


**Supplementary Figure S2. Changes of lipid peroxidation and antioxidant enzymes activities following melatonin treatment for 12 mo.** (a): Malondialdehyde (MDA) content. (b-d): Activities of total antioxidant capacity (T-AOC) glutathione peroxidase (GSH-Px) and glutathione reductase (GSH-Rd). Data are means ± SD (n = 7/group). ^*^P < 0.05; ^**^P < 0.01 versus the Young group. ^##^p < 0.01 versus the Vehicle group.


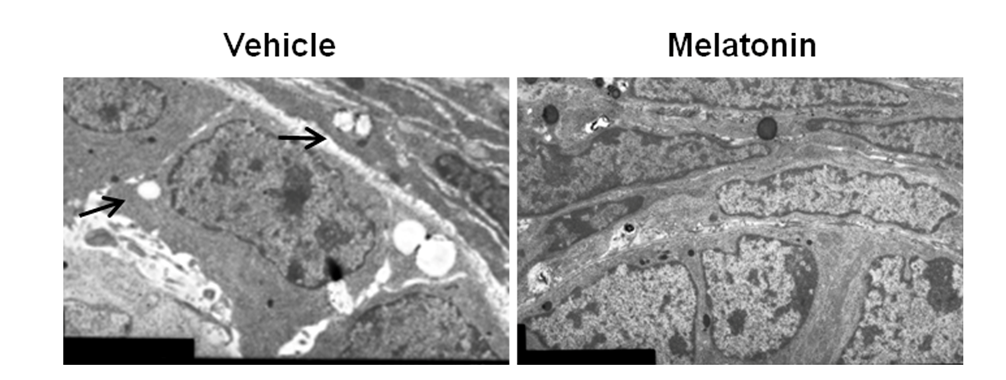


**Supplementary Figure S3. Representative electron micrographs showing ultrastructural details of granulosa cells after mice treated with vehicle or melatonin for 12 mo.** Black arrow: Defective mitochondria (magnified × 10,000 times).


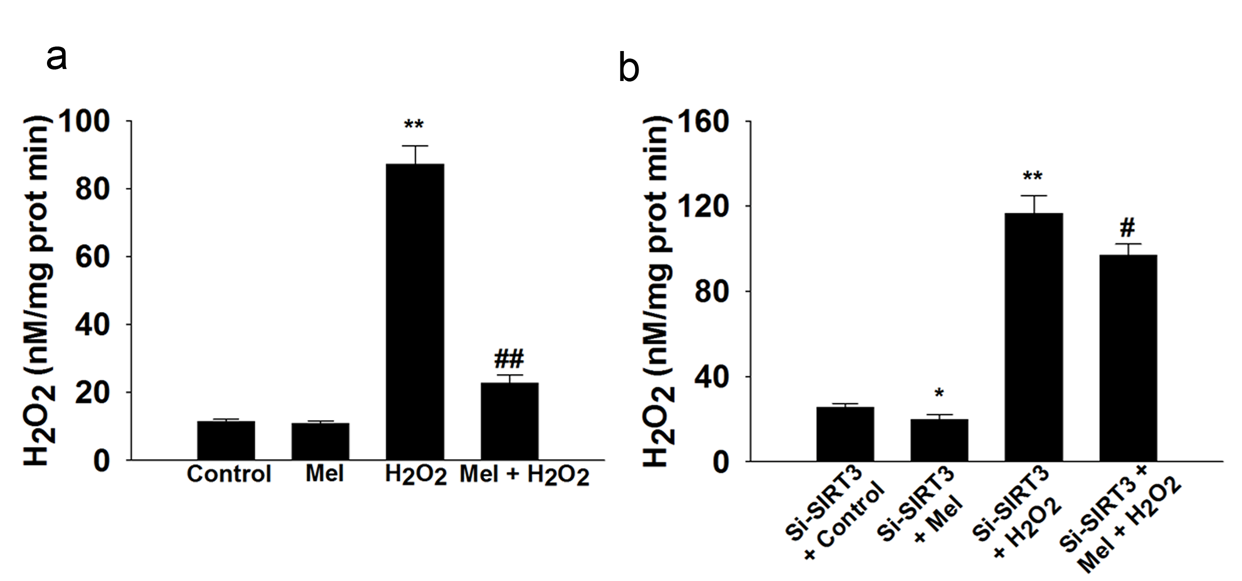


**Supplementary Figure S4. Effects of melatonin on the generation of** **H_2_O_2_ in granulosa cells.** (a): Melatonin treatment significantly inhibited the generation of H_2_O_2_. (b): SIRT3 siRNA treatment significantly attenuated the beneficial effects of melatonin on eliminating ratio of H_2_O_2_. All values are the mean ± SD of the results from three independent experiments. ^*^p<0.05; ^**^p<0.01 versus the Control group. ^#^p < 0.05; ^##^p < 0.01 versus the H_2_O_2_ group.
